# Statin inhibits large hepatitis delta antigen-Smad3 -twist-mediated epithelial-to-mesenchymal transition and hepatitis D virus secretion

**DOI:** 10.1186/s12929-020-00659-6

**Published:** 2020-05-21

**Authors:** Yuh-Jin Liang, Cheng-Pu Sun, Yu-Chen Hsu, Yi-Wen Chen, I-An Wang, Chien-Wei Su, Mi-Hua Tao, Jaw-Ching Wu

**Affiliations:** 1 Translational Research Division, Medical Research Department, Taipei Veterans General Hospital, No.201, Sec. 2, Shipai Rd., Beitou District, Taipei, 11217 Taiwan, Republic of China; 2 Institute of Biomedical Sciences, Academia Sinica, 128 Academia Road, Section 2, Nankang, Taipei, 115 Taiwan, Republic of China; 3 Institute of Clinical Medicine, National Yang-Ming University, No.155, Sec.2, Linong Street, Taipei, 112 Taiwan, Republic of China; 4 Division of Gastroenterology, Department of Medicine, Taipei Veterans General Hospital, No.201, Sec. 2, Shipai Rd., Beitou District, Taipei, 11217 Taiwan, Republic of China; 5 Faculty of Medicine, School of Medicine, National Yang-Ming University, No.155, Sec.2, Linong Street, Taipei, 112 Taiwan, Republic of China; 6 Cancer Progression Research Center, National Yang-Ming University, No.155, Sec.2, Linong Street, Taipei, 112 Taiwan, Republic of China

**Keywords:** Prenylation inhibitors, Statins, Hepatitis delta antigens, Smad3, *Twist* promoter, Epithelial-mesenchymal transition

## Abstract

**Background:**

Hepatitis D virus (HDV) infection may induce fulminant hepatitis in chronic hepatitis B patients (CHB) or rapid progression of CHB to cirrhosis or hepatocellular carcinoma. There is no effective treatment for HDV infection. HDV encodes small delta antigens (S-HDAg) and large delta antigens (L-HDAg). S-HDAg is essential for HDV replication. Prenylated L-HDAg plays a key role in HDV assembly. Previous studies indicate that L-HDAg transactivates transforming growth factor beta (TGF-β) and induces epithelial-mesenchymal transition (EMT), possibly leading to liver fibrosis. However, the mechanism is unclear.

**Methods:**

The mechanisms of the activation of *Twist* promoter by L-HDAg were investigated by luciferase reporter assay, chromatin immunoprecipitation, and co-immunoprecipitation analysis. ELISA and Western blotting were used to analyze L-HDAg prenylation, TGF-β secretion, expression of EMT markers, and to evaluate efficacy of statins for HDV treatment.

**Results:**

We found that L-HDAg activated Twist expression, TGF-β expression and consequently induced EMT, based on its interaction with Smad3 on Twist promoter. The treatment of statin, a prenylation inhibitor, resulted in reduction of *Twist* promoter activity, TGF-β expression, and EMT, and reduces the release of HDV virions into the culture medium.

**Conclusions:**

We demonstrate that L-HDAg activates EMT via Twist and TGF-β activation. Treatment with statins suppressed Twist expression, and TGF-β secretion, leading to downregulation of EMT. Our findings clarify the mechanism of HDV-induced EMT, and provide a basis for possible novel therapeutic strategies against HDV infection.

## Background

Hepatitis D virus (HDV) infection may induce fulminant hepatic failure or aggravate underlying chronic hepatitis B to liver cirrhosis, liver failure, or hepatocellular carcinoma (HCC); alternatively, it may display a slow, subclinical course [1–3]. The molecular mechanisms underlying this variety of clinical manifestations and outcomes remain poorly understood. HDV is a defective satellite virus whose assembly requires a supply of hepatitis B virus surface antigen (HBsAg) from hepatitis B virus (HBV) [4]. HDV encodes delta antigens (HDAg), which have two isoforms: small delta antigens (S-HDAg) and large delta antigens (L-HDAg) [4, 5]. S-HDAg is involved in transactivation of HDV RNA replication, while prenylated L-HDAg plays a key role in packaging of complete HDV virions through its interaction with S-HDAg, HDV RNA, and HBsAg [4, 5]. HDV viruses have been divided into at least eight major clades based on their genome diversity: HDV-1 to HDV-8 [6]. HDV-1 is distributed worldwide, while HDV-2 and HDV-4 are restricted to certain Far Eastern regions such as Taiwan, Japan, and Yakutia [6–9].

Disease outcomes are determined by HDV genotypes [7, 8], HBV and/or HDV viral loads, HBsAg levels and sequences [3, 7–10], and other confounding factors such as transforming growth factor-β (TGF-β) levels [10]. TGF-β plays important roles in liver fibrosis and cirrhosis [11]. Choi et al. reported that L-HDAg may induce liver fibrosis through TGF-β-induced signal transduction [12]. Activation of specific receptors by TGF-β induces epithelial-mesenchymal transition (EMT) in many types of epithelial cells in culture [13]. Enhanced TGF-β signaling has been implicated as a key effector of EMT in cancer progression and metastasis by several lines of study, and TGF-β is therefore considered a master positive regulator of EMT. When injury and inflammation persist, EMT generates fibroblastic cells that accumulate and cause progressive fibrosis [14]. The EMT process is characterized by declining levels of epithelial cell-specific proteins (e.g., E-cadherin) and increasing levels of mesenchymal cell-specific proteins (e.g., α-smooth muscle actin, vimentin, collagen) [14]. We demonstrated previously that expression of transcription factors Twist and Snail in HCC is associated with EMT, and with recurrence of HCC following tumor resection [15].

Sustained virological and biochemical remission rates are still low in chronic hepatitis D patients treated by interferon. Nucleoside and nucleotide analogues are effective for suppressing HBV replication, but ineffective for suppressing HDV replication [16]. Assembly of HDV virus-like particles and of complete, infectious HDV virions of genotypes I and III was blocked by the farnesyltransferase-inhibitory compounds BZA-5B and FTI-277 [17, 18]. These studies suggest potential application of farnesyltransferase inhibitors in targeting of HDV assembly. Statins, a class of drugs widely used for treatment of hypercholesterolemia, inhibit the rate-limiting enzyme in the cholesterol biosynthetic pathway, 3-hydroxy-3-methylglutaryl-coenzyme A (HMG-CoA) reductase, and indirectly decrease levels of biologically intermediate substrates for prenylation [19, 20]. The isoprenoids geranylgeranyl pyrophosphate (GGPP) and farnesyl pyrophosphate (FPP) are added to C-termini of the Ras superfamily of small G-proteins (e.g., Rho, Rab). Isoprenoid modification is essential for facilitating GTPase interactions with cytoplasmic regulators, cellular membranes, and effectors [19]. Alteration of Rho GTPase signaling plays important roles in both initiation and progression of HCC. Rho-dependent pathways promote cancer cell migration and metastasis [21].

In the present study, we found that L-HDAg activated Twist expression, TGF-β expression and consequently induced EMT. On the other hand, statin treatment resulted in reduction of *Twist* promoter activity, TGF-β expression, and EMT. Our findings help clarify the mechanisms of HDV-induced EMT, and provide a basis for future improvement of chronic hepatitis D therapy.

## Materials and methods

### Cell culture, transfection, and treatment with statins

Human HCC cell line Huh7 was obtained from American Type Culture Collection (ATCC; USA) and maintained in Dulbecco’s modified Eagle’s medium (DMEM) (Gibco; USA) containing 10% fetal bovine serum (FBS), 1% non-essential amino acid, 1% L-glutamine, and 1% penicillin-streptomycin (Gibco) in a humidified incubator (Thermo Fisher; USA) at 37 °C under 5% CO_2_ atmosphere. For transfection, cells were plated on cell culture dishes at 70% confluence, and transfected on the following day with FuGENE HD transfection reagent (Roche; Switzerland). For treatment with statins, the HMG-CoA-reductase inhibitors fluvastatin (Sigma-Aldrich; USA), simvastatin (Sigma-Aldrich), atorvastatin (Sigma-Aldrich), rosuvastatin (Astra Zeneca; London, UK), lovastatin (Sigma-Aldrich), and pravastatin (Sigma-Aldrich) were dissolved in DMSO, and cells were treated with each of the six types of statin (final concentration 5 or 25 μM by dilution in medium) for three or 9 days and subjected to luciferase reporter assay, enzyme-linked immunosorbent assay (ELISA), and Western blotting. DMSO alone was used as negative control.

### Plasmid construction for full genome of HDV, HBV, L-HDAg, S-HDAg, and prenylation-deficient C211S mutant of L-HDAg

Plasmids expressing full genome of HDV, HBV, three genotypes of L-HDAg- and S-HDAg were isolated and constructed as described previously [9, 10]. The sources of the HBV and HDV plasmids were derived from CHD patients. Plasmids expressing full genome of HDV, HBV, three genotypes of L-HDAg- and S-HDAg were isolated and constructed as described in our previously publications [2, 8]. In this study, two-copy of HDV genome containing plasmid, were used for HDV genome replication. The HDV genomic sequences, named TWD2577–66 are available in GenBank with accession numbers AF425644. The plasmid contains 1.46x HBV genome were used for HBV genome replication. The accession numbers of GenBank for HBV genomic sequences were EF494377.

For cloning of HDAg, pHDV-D2G was digested with XbaI/ SphI, and HDAg fragment was isolated and subcloned into XbaI/SphI-digested pCMV-EBNA (Clontech Laboratories; USA). Plasmids expressing three genotypes of L-HDAg with single-residue substitution of cysteine by serine at amino acid (aa) 211 were constructed using QuikChange II site-directed mutagenesis kit (Agilent Technologies; USA). Primer sequences for site-directed mutagenesis are listed in Table 1.

**Table 1:** Primer sequences for plasmid construction of SBE mutation of *Twist* promoter

Primer	Sequence
pXP2-Twist-SBE site1-mt-forward	5′-GGAGGTATAAGAGCCTCCAA**TTGC**GCAGCTCTCGCCCA-3’
pXP2-Twist-SBE site1-mt-reverse	5′-TGGGCGAGAGCTGC**GCAA**TTGGAGGCTCTTATACCTCC-3’
pXP2-Twist-SBE site2-mt-forward	5′-CAGCTCTCGCCCAACTCC**AGCCA**ACCTCGCGGGCTCTGCAG-3’
pXP2-Twist-SBE- site2-mt-reverse	5′-CTGCAGAGCCCGCGAGGT**TGGCT**GGAGTTGGGCGAGAGCTG-3’

Boldface + underlining: mutated nucleotides for disruption of SBE for Smad3 binding

**Fig. 1:**
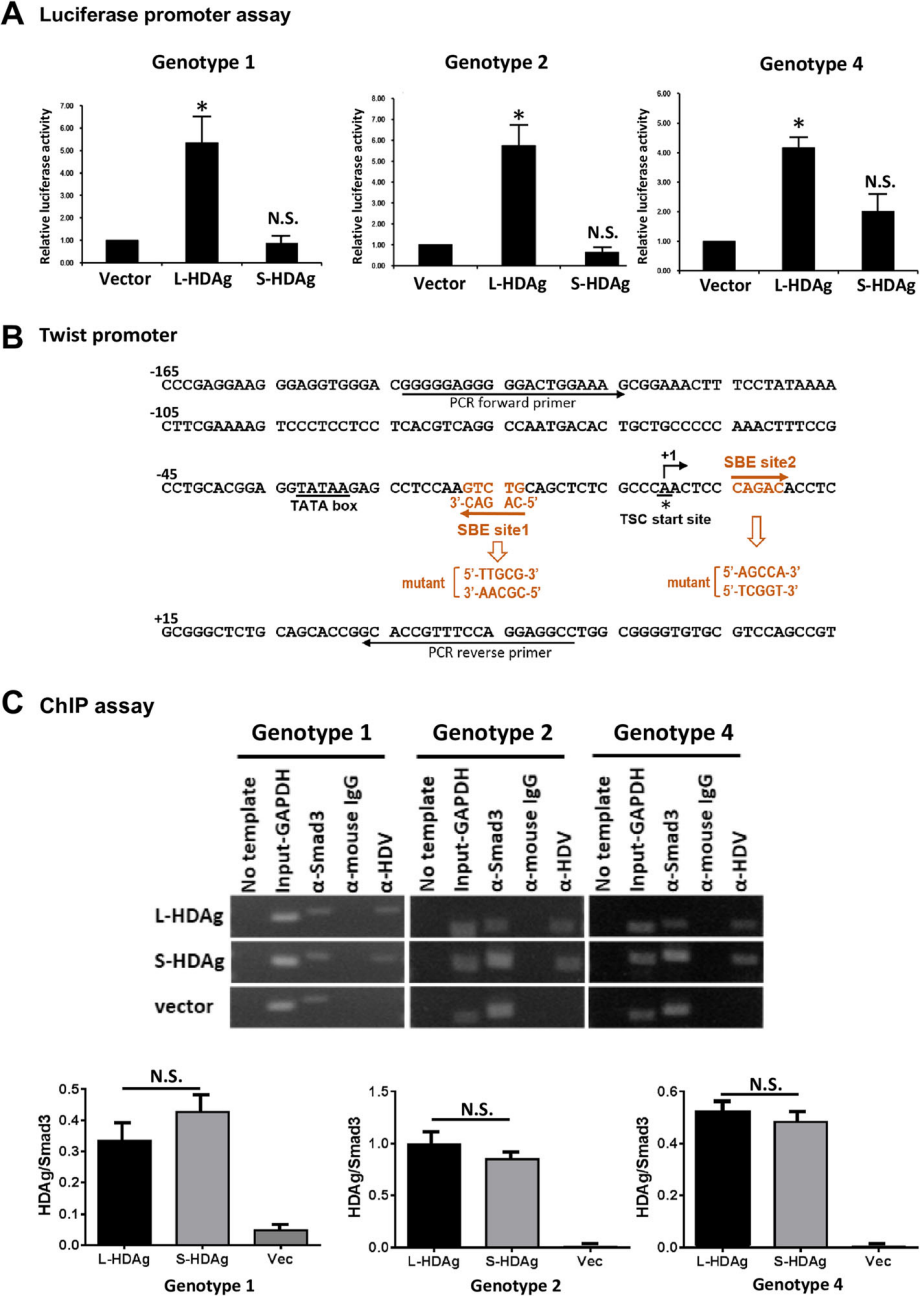
L-HDAg activates *Twist* promoter through binding with Smad3 on Smad binding elements (SBEs). **a** Huh7 cells were co-transfected with *Twist* promoter reporter pXP2-Twist with either L-HDAg- or S-HDAg-expressing plasmids of three genotypes. The pSV-β-galactosidase-expressing plasmid was co-transfected for monitoring transfection efficiencies. Luciferase activity was measured and normalized with β-galactosidase activity value. The fold change of luciferase activity relative to β-galactosidase activity were expressed as mean ± SD from three independent experiments. *: *p* < 0.05 (Student’s t-test), N.S.: no significant differences compared to Vector control. **b** Nucleotide sequence of proximal *Twist* promoter. + 1: principal transcription start site of *Twist* gene. Underlining: TATA box sequence. Notations below sequence: two potential Smad-binding sites (SBE site1 and site2) and mutated sequence of Smad-binding region. Underlining with solid arrow: position of forward or reverse primer for PCR amplification of ChIP. **c** Huh7 cells expressing L-HDAg or S-HDAg of three genotypes were chromatin IP’d with mouse anti-HDAg monoclonal Ab, anti-Smad3 Ab (positive control), or anti-mouse IgG (negative control). ChIP-enriched DNA samples were analyzed by PCR using Smad3 binding element (SBE)-specific primers with amplicon 196-bp. The original input was amplified by PCR with GAPDH promoter specific primers (Input-GAPDH, 166 bp). Densitometric analysis were indicated. Results shown are mean ± SD from five independent experiments. N.S.: no significant differences

WT sequence of *Twist* promoter containing SBE consensus sequence of CAG(AC)(CC) is shown in Fig. 1b

### Plasmid construction for luciferase reporter assay

The *Twist* promoter region (spanning from − 139 to + 48 bp relative to transcription start site of *Twist* gene) was cloned and inserted into pXP2 luciferase reporter vector to generate pXP2-Twist [22]. Two Smad binding elements (SBEs) composed of conserved CAGACA sequences in *Twist* promoters were mutated with QuikChange II kit as above. Primer sequences used for site-directed mutagenesis are listed in Table 2. Sequence of *Twist* promoter containing SBEs with consensus sequence CAG (AC)|(CC) is shown in Fig. 1b.

**Table 2:** Primer sequences for plasmid construction of SBE mutation of *Twist* promoter

Primer	Sequence
pXP2-Twist-SBE site1-mt-forward	5′-GGAGGTATAAGAGCCTCCAA**TTGC**GCAGCTCTCGCCCA-3′
pXP2-Twist-SBE site1-mt-reverse	5′-TGGGCGAGAGCTGC**GCAA**TTGGAGGCTCTTATACCTCC-3′
pXP2-Twist-SBE site2-mt-forward	5′-CAGCTCTCGCCCAACTCC**AGCCA**ACCTCGCGGGCTCTGCAG-3′
pXP2-Twist-SBE- site2-mt-reverse	5′-CTGCAGAGCCCGCGAGGT**TGGCT**GGAGTTGGGCGAGAGCTG-3′

Boldface + underlining: mutated nucleotides for disruption of SBE for Smad3 binding

WT sequence of *Twist* promoter containing SBE consensus sequence of CAG(AC)(CC) is shown in Fig. 1b

### Smad3-knockdown cell lines

A shSmad3 clone containing short-hairpin (sh) targeted Smad3 was from National RNAi Core Facility (Taipei, Taiwan). Lentivirus production was performed using a HEK293T cell viral packaging system. Huh7 cells were transduced with Smad3-sh sequence containing lentivirus with multiplicity of infection (MOI) = 2. Stable clones were selected with puromycin (1 μg/ml). Antibiotic-resistant clones were pooled to avoid clonal variation.

### Western blotting analysis

Cells were lysed in lysis buffer (20 mM Tris-HCl [pH 7.4], 137 mM NaCl, 1 mM EDTA [pH 8.0], 1% Triton X-100, 2 mM sodium pyrophosphate, 1 mM Na_3_VO_4_) with 1 mM PMSF, phosphatase inhibitors (Roche), and cocktail protease inhibitors (Sigma-Aldrich). The lysate was incubated on ice for 30 min and cleared by centrifugation at 13,000 x *g* for 20 min at 4 °C. Protein samples were separated by SDS-PAGE and blotted on PVDF membranes. Blots were probed with specific primary Abs, then incubated with appropriate HRP-conjugated secondary Ab for 1 h. Bands were visualized with ECL reagents (PerkinElmer; USA). Quantification of bands intensities was performed using ImageJ (NIH, USA) and Alpha Imager 3400 (Alpha Innotech, USA).

Primary Abs used for immunoblotting were directed to: Snail (1:500; C15D3, Cell Signaling Technology; USA), Twist (1:100; ab50881, Abcam; UK), E-cadherin (1:1000; R868, Bioworld Technology; USA), vimentin (1:1000; V6630, Sigma-Aldrich), and heat shock protein 70 (Hsp70; 1:5000; B6, Santa Cruz Biotechnology; USA). For analysis of HDAg expression, blots were probed with anti-HDV-positive human serum (1:5000) or monoclonal antibody against HDAg (Binding Site, MC406.3). Secondary Abs used were HRP-conjugated sheep anti-mouse IgG (1:5000; ab6808–1, Abcam) and goat anti-rabbit IgG (1:5000; 111–035-003, Jackson Laboratories; USA).

### Luciferase reporter assay

The pXP2-Twist reporter plasmid was co-transfected into Huh7 cells with plasmids expressing L-HDAg, S-HDAg, or prenylation-deficient L-HDAg mutant. To monitor transfection efficiency, a plasmid expressing bacterial β-galactosidase gene (pCMV-β gal) was co-transfected as internal control in each experiment. Cells were harvested 72 h after transfection, and luciferase assay was performed using Luciferase Reporter Assay System (Promega; USA) per the manufacturer’s instructions. Relative promoter activities were expressed as fold change in luciferase activities after normalization relative to β-galactosidase activity value.

### Chromatin immunoprecipitation assay

Chromatin was immunoprecipitated with anti-HDAg Ab (mouse monoclonal antibody against HDAg; Binding Site, MC406.3) and analyzed by conventional PCR with ChIP primers. Cells were grown in culture dishes, cross-linked with 1% formaldehyde for 10 min at room temperature, added with 0.125 M glycine to quench unreacted formaldehyde, washed with ice-cold PBS, and lysed with SDS cell lysis buffer. Fixed chromatins were broken down to ~ 500–200 bp by sonication. Cell lysates were immunoprecipitated (IP’d) with mouse anti-HDAg Ab. The mouse IgG and anti-Smad3 Ab were used as negative and positive controls. Immunocomplexes were incubated 1 h at 4 °C with gentle rotation, adsorbed with protein G-agarose overnight at 4 °C with gentle rotation, washed sequentially with low-salt wash buffer, high-salt wash buffer, LiCl wash buffer, and TE buffer, eluted with 1% SDS and 0.1 M NaHCO_3_, and subjected to decrosslinking overnight at 65 °C. Genomic DNA fragment in the antibody-adsorbed complex was purified by proteinase K digestion and phenol/ chloroform extraction, subjected to conventional PCR reactions to amplify response elements with specific primers. Primers for amplifying fragment containing two SBEs were 5′-GGGGGAGGGGGACTGGAAAG-3′ (forward) and 5′-GGCCTCCTGGAAACGGTGC − 3′ (reverse), resulting in a 196-bp fragment. Sequence of *Twist* promoter region with SBE sites is shown in Fig. 1b.

### Co-immunoprecipitation (co-IP) assay

Huh7 cells were lysed in NET buffer (50 mM Tris-HCl [pH 7.0], 5 mM EDTA, 150 mM NaCl, 0.5% Nonidet P-40) with phosphatase inhibitors and cocktail protease inhibitors. Lysate was incubated on ice for 30 min, and cleared by centrifugation at 13,000 x *g* for 30 min at 4 °C. IP was performed with rabbit polyclonal Smad3 Ab (Abcam, ab28379) in the presence of protein A Sepharose (Dynabeads Protein A, #10002D, Invitrogen; USA) for 2 h at 4 °C in a rocking incubator. The Smad3 antibody from Abcam (ab28379) was previously validated using Smad3 KO animals and the data have been published [23]. Resulting immunocomplexes were subjected to immunoblotting. Blots were probed with anti-HDV-positive human serum to detect L-HDAg or S-HDAg, then incubated with HRP-conjugated goat anti-human IgG + IgM secondary Ab for 1 h. Bands were visualized with ECL reagents (PerkinElmer).

### Northern blot assay

Total cellular RNAs from HBV-HDV co-transfected Huh-7 cells were extracted by TRIzol reagent (Life Technologies, Grand Island, NA). RNA purification was performed according to the manufacturer’s instructions. A total of 20 microgram of RNA was analyzed by Northern blotting as previously described [10]. After fixation by UV illumination, RNA was hybridized with digoxigenin (DIG)-labeled cDNA probes derived from different genotypes of HDV. Hybridization was performed with DIG labeling and detection Kit (Roche Diagnostics System, Basel, Switzerland) at 55 °C overnight. The fragment of the glyceraldehyde-3-phosphate dehydrogenase GAPDH-cDNA was used as a control probe.

### Real-time RT-PCR for HDV RNA quantitative assay

The absolute quantification of HDV RNA were accomplished by using the standard curve method as our previously publication described [9, 10]. HDV genome coding partial HDAg was amplified and inserted into the pCRII vector as the standards. By using a series of dilutions of previously titrated standard plasmids ranging from 5 to 5 × 10^6^ copies in triplicate, the standard curve was created. The mean cycle threshold (CT) values of unknown samples were compare with the standard curves and infer the HDV copy number. In this study, total cellular RNAs from HBV-HDV co-transfected Huh-7 cells were extracted by Viral DNA/RNA Mini Kit (Novelgene, NV-S050). The synthesis of cDNA was prepared using SuperScript™ III Reverse Transcriptase (Invitrogen, 18,080,085). Real-time PCR was performed by using the TaqMan™ Universal Master Mix II, no UNG (TaqMan, 4,440,047) and TaqMan MGB HDVII probe (TaqMan, 4,316,032) with HDV specific primer pairs: 5′-TCg TCT TCA ACg gTC AAC CT-3′ and 5′-AAg gAA ggC CCT CgA gAA CA-3′. The correlation coefficients were repeatedly 0.995, and the slopes were ranged from 3.1 to 3.4, the linearity of quantification ranged from 2 × 10^3^ to 2 × 10^9^ copies/ml.

### Quantitative analysis of HBsAg and HBV DNA

HBsAg expression was measured by ELISA kit (Elecsys HBsAg II; Cobas) and HBV DNA expression was measured by TaqMan HBV Test, V. 2.0 (Cobas). The detailed procedure were performed as previously described [9].

### Quantification analysis of TGF-β

Huh7 cells transfected with L-HDAg- or S-HDAg-expressing plasmids were incubated in DMEM supplemented with 2% FBS, and supernatants were collected after 3 days. Total TGF-β in culture supernatants was activated by adding 20 μl of 1 N HCl per 100 μl culture medium for 10 min, neutralized by adding 20 μl of 1.2 N NaOH/ 0.5 M HEPES, and detected using Human TGF-beta 1 Quantikine ELISA Kit (DB100B, R&D Systems; USA) per the manufacturer’s instructions.

### Statistical analysis

Data were analyzed by one-way ANOVA followed by Newman-Keuls multiple comparison post hoc test to compare all groups with control group, or by unpaired Student’s t-test to compare designated pairs of groups, using Prism 5 software program (GraphPad). Differences were considered significant at *p* < 0.05.

## Results

### L-HDAg activates twist promoter

Huh7 cells were transiently co-transfected with HDAg-expressing plasmid and *Twist* promoter-driven luciferase reporter plasmid, pXP2-Twist. Cells were harvested 72 h post-transfection, and luciferase activities were measured. Regardless of which HDV genotype (1, 2, or 4) antigen was transfected, ectopic expression of L-HDAg increased *Twist* promoter activity 4- to 6-fold relative to vector control (Fig. 1a). In contrast, S-HDAg had no effect on *Twist* promoter activity.

Both L-HDAg and S-HDAg were shown to interact with Smad3 transcription factors in vitro and in vivo [12]. Analysis of *Twist* promoter sequence using MatInspector [24] revealed two putative Smad3 sites clustered at the proximal promoter region with consensus SBE of CAG(AC)|(CC) (Fig. 1b). To test the possibility that HDAg activates *Twist* promoter by binding to Smad3 at SBEs, we performed chromatin immunoprecipitation (ChIP) assays. Huh7 cells were transfected with either L-HDAg- or S-HDAg-expressing plasmid, and binding of L-HDAg to clustered SBE sites of *Twist* promoter were detected by ChIP assay using antiserum from delta antigen hepatitis patients. Regardless of which HDV genotype antigen was transfected, L-HDAg and S-HDAg showed binding to *Twist* promoter at positions corresponding to SBEs (Fig. 1c). In contrast, anti-mouse IgG had no effect on SBE sites of *Twist* promoter. With Smad3 specific antibody enrichment, binding of Smad3 to SBEs was detected as positive control (Fig. 1c, Anti-Smad3). DNA containing cell lysate was aliquoted and directly PCR amplified using GAPDH-specific primer pairs, as input sample control (Fig. 1c, Input-GAPDH). In quantification the ChIP result, the level of SBEs direct enriched with Smad3 antibody were used as the denominator and the level of SBEs enriched with L- or S-HDAg antibody as the numerator. We found that the enrichment of SBEs on Twist promoter by L-HDAg or S-HDAg antibody were no significant difference (Fig. 1c, bar-graphs).

**Fig. 2:**
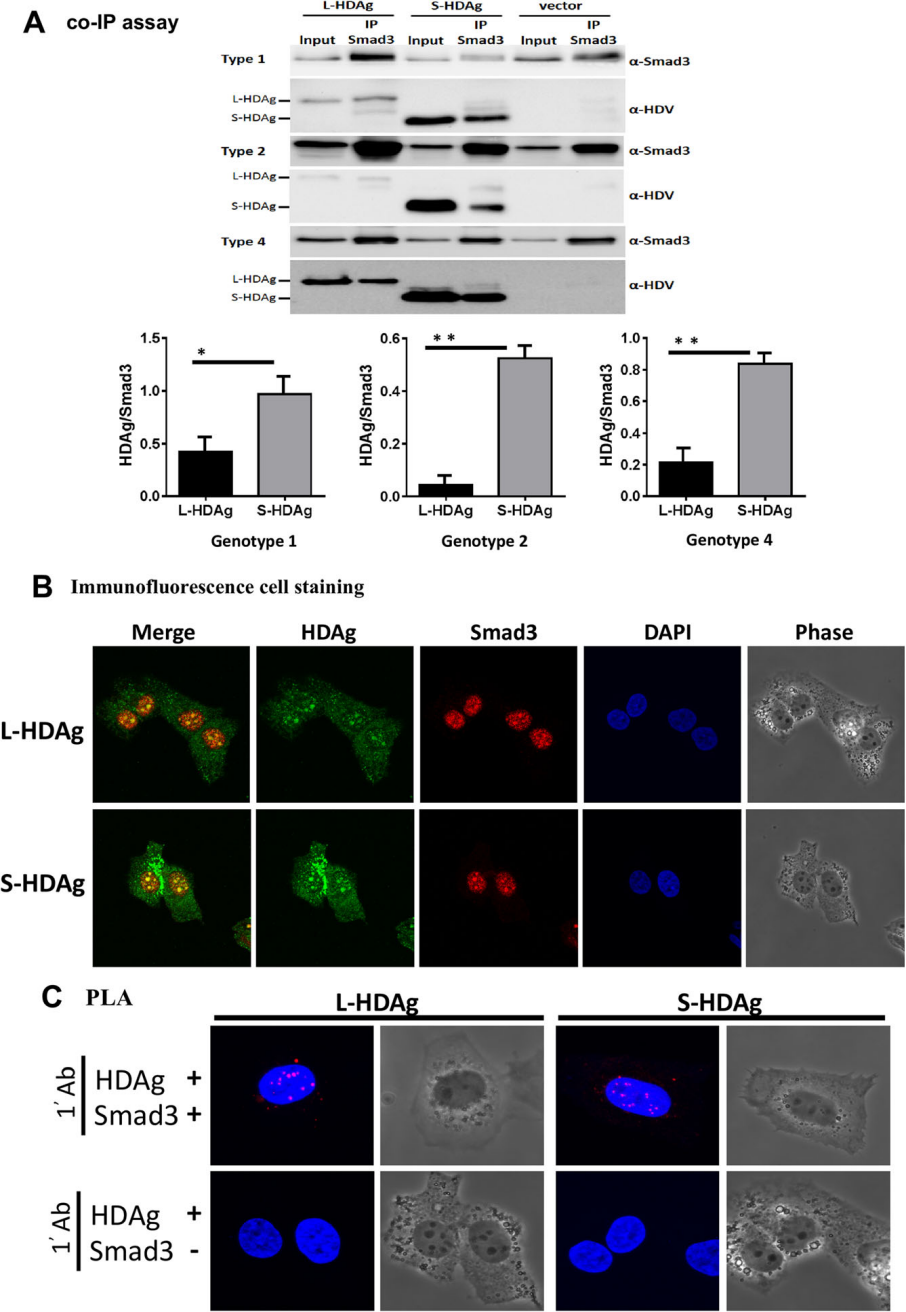
Interaction of HDAg and Smad3. **a** Huh7 cells expressing L-HDAg or S-HDAg of three genotypes were IP’d with anti-Smad3 Ab, and the IP’d lysates were subjected to SDS-PAGE and immunoblotting with antiserum from delta antigen hepatitis patients or with anti-Smad3 Ab. Densitometric analysis were indicated. Results shown are mean ± SD from five independent experiments. *: *p* < 0.05; **p < 0.01. **b** Colocalization analysis of L-HDAg or S-HDAg (green) and the Smad3 (red) using double-immunofluorescence staining. Detection by monoclonal antibody against HDAg, both L-HDAg and S-HDAg (green) showed a high degree of colocalization with Smad3 (red), particularly in the nucleoli region. **c** L-HDAg or S-HDAg associations with Smad3 were investigated by in situ proximity ligation assay (PLA). Each PLA signal is visualized as a red fluorescent spot, and represents one detected association event between HDAg and Smad3. Incubation of HDAg antibody alone was a negative control. Cell nuclei were stained with DAPI (blue), corresponding phase contrast images are also shown

Smad3/ HDAg interaction was further evaluated using co-IP assays. Lysates from Huh7 cells transfected with L-HDAg or S-HDAg were IP’d with anti-Smad3 Ab, and co-IP’d proteins were detected by Western blotting analysis using anti-HDAg-positive human serum. Regardless of which HDV genotype antigen was transfected, both S-HDAg and L-HDAg were co-IP’d by Smad3 (Fig. 2a). In quantification of the co-IP result, the direct immuno-precipitated Smad3 were serves as the denominator and co-IP of L- or S-HDAg by Smad3 antibody were serves as the numerator. We found that the co-IP of S-HDAg by Smad3 antibody was much abundant than co-IP of L-HDAg by Smad3 antibody (Fig. 2a, panels of bar-graphs).

The association of HDAg and Smad3 were further investigated by double-immunofluorescence staining. Consistent with co-IP results, both L-HDAg and S-HDAg (green) showed a high degree of colocalization with Smad3 (red), particularly in the nucleoli region (Fig. 2b). In situ interactions of HDAg and Smad3 were investigated by a proximity ligation assay (PLA). In this technique, when a pair of PLA probes binds two molecules that are in close proximity (< 16 nm), complementary DNA strands conjugated to PLA probes are ligated, amplified, and visualized as distinct spot using a fluorescent probe. The association of PLA signals detecting with antibodies of HDAg and Smad3 were observed in both L-HDAg and S-HDAg transfected cells. In addition, treatment with the HDAg antibodies alone did not give a PLA signal (Fig. 2c). Taken together, our results indicate that both S-HDAg and L-HDAg bind to Smad3 protein on SBEs of proximal *Twist* promoter region.

### Twist promoter activation by L-HDAg is reduced when its SBE is disrupted or in the absence of Smad3

**Fig. 3:**
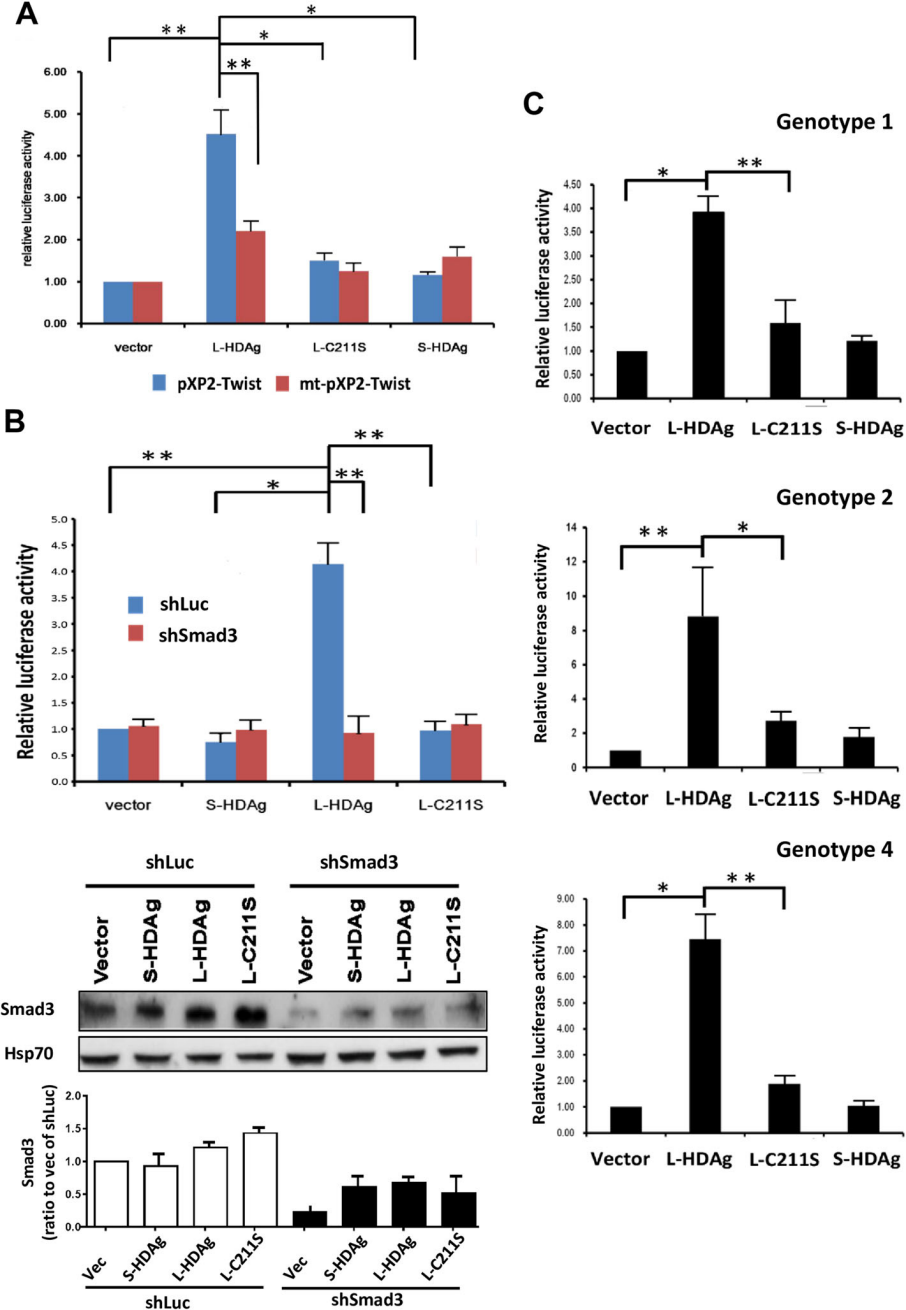
Activation of Twist promoter by L-HDAg was strongly reduced by disruption of SBEs, C211S mutation of L-HDAg, or Smad3 knockdown. **a** Wild type Twist promoter reporter (pXP2-Twist) or SBEs mutated reporter (mt-pXP2-Twist) were co-transfected into Huh7 cells with plasmids encoding L-HDAg, C211S mutant of L-HDAg (L-C211S), S-HDAg, or pcDNA control vector. The fold change of luciferase activity relative to β-galactosidase activity were shown as mean ± SD from three independent experiments. *: *p* < 0.05; **p < 0.01 (Student’s t-test). **b** Effect of Smad3 knockdown on Twist promoter activity, by luciferase assay. Plasmids expressing Smad3-targeting shRNA (shSmad3) and non-targeting control (shLuc) were transiently transfected with pXP2-Twist and L-HDAg, L-C211S, S-HDAg, or pcDNA control plasmids. Transfectants lysate were analyzed by luciferase assay (left panel) or immunoblotting (right panel) with anti-Smad3 Ab, antiserum from delta antigen hepatitis patients, or anti-Hsp70 Ab as internal control. *: *p* < 0.05 (compared with vector control in shLuc group). **c** Prenylation-deficient mutants of L-HDAg (C211S) from all three genotypes lost the ability to activate Twist promoter. The pXP2-Twist reporter was co-transfected with L-HDAg, L-C211S, S-HDAg, or pcDNA3 control plasmids, and transfectants were analyzed by luciferase assay

To further evaluate relationships among L-HDAg, SMAD3, and *Twist* promoter, two putative SBEs in *Twist* promoter were disrupted by site-directed mutagenesis (Fig. 1b). Huh7 cells were transiently co-transfected with wild-type (WT) or mutant *Twist* promoter reporter construct (pXP2-Twist vs. mt-pXP2-Twist) and HDAg-expressing plasmid. After 72 h, cell lysates were subjected to luciferase reporter assay (Fig. 3a). In L-HDAg transfected cells, the mutations of the SBE motifs in the Twist promoter showed a significant decrease in luciferase activity of approximately 59% compared to wild-type Twist promoters. In contrary, cells transfected with C211S mutant of L-HDAg (L-C211S), S-HDAg, or pcDNA3 control vector were unable to activate Twist promoter and did not show significant differences in luciferase activity (Fig. 3a).

The concept that L-HDAg activates *Twist* promoter by interacting with Smad3 was further evaluated by measuring *Twist* luciferase reporter activity in Smad3 knockdown cells. Smad3 was knocked down using a lentivirus-based expression vector carrying shSmad3, obtained from the RNAi core facility of Academia Sinica. Following puromycin selection, cell lines of shSmad3 and non-targeting shLuc control were established from Huh7. Huh7-sh*Smad3* cells were then transiently transfected with either L-HDAg- or S-HDAg-expressing plasmids. Lysates were collected 72 h post-transfection for Western blotting and luciferase assays. Western blotting results indicated that Smad3 level was significantly reduced in shSmad3-expressing cells relative to non-targeting shLuc control cells (Fig. 3b). In shSmad3-expressing cells, the L-HDAg showed a significant decrease in Twist transactivation activity of approximately 70% compared to shLuc-expressing control cells (Fig. 3b). *Twist* promoter activity was not significantly altered when S-HDAg-expressing plasmid was transfected into Huh7 cells, consistently with results shown in Fig. 1. These findings indicate that L-HDAg activates *Twist* promoter through interaction with Smad3.

### Activation of twist promoter by L-HDAg is dependent upon the C-terminal prenylation domain

To investigate the possibility that prenylation at Cys-211 is also necessary for activation of *Twist* promoter, we substituted serine for cysteine at this position to create prenylation-deficient mutant C211S. In genotypes 1, 2 and 4, the C211S mutants of HDAgs showed a significant decrease in Twist promoter activity of approximately 63, 78 and 74% compared to wild type HDAg (Fig. 3c). Accordingly, no changes in luciferase activity with *Twist* promoter mutation or Smad3 knockdown were observed in C211S mutants (Fig. 3a, b).

**Fig. 4:**
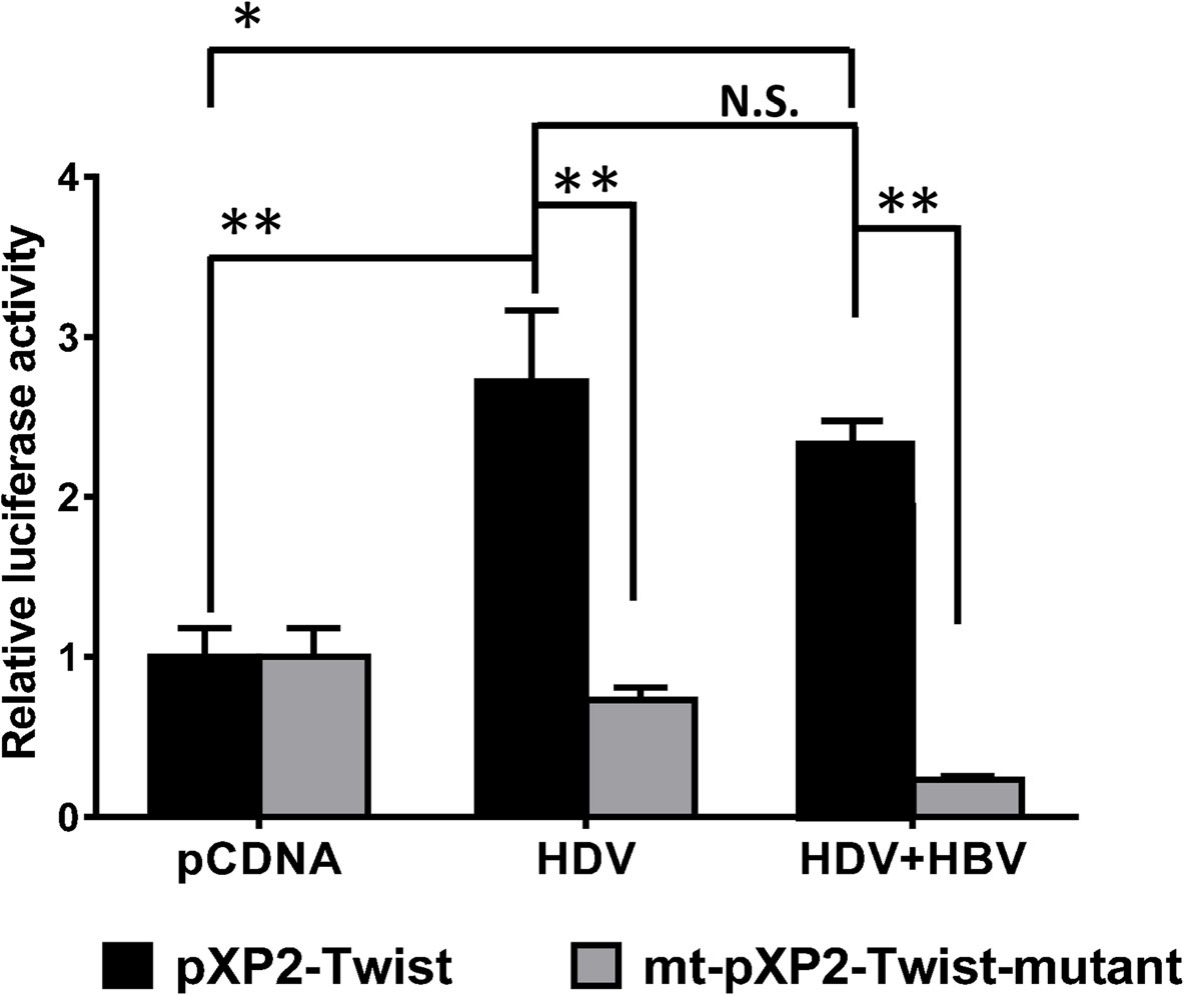
Twist promoter activated in HBV/HDV co-transfected Huh7 cells. Wild type *Twist* promoter reporter (pXP2-Twist) or SBEs mutated reporter (mt-pXP2-Twist) were co-transfected with whole genome of HDV and HBV plasmids as indicated. Luciferase activity was measured using the dual-luciferase reporter assay system with Renilla luciferase activity as internal control. The fold change of luciferase activity relative to Renilla activity were shown as mean ± SD from three independent experiments. *: *p* < 0.05; **p < 0.01, N.S.: no significant differences. (Student’s t-test)

In order to determine that HDAg activated Twist promoter does not only occur in L-HDAg overexpression system, we co-transfected whole genome of HDV and HBV in Huh7 cells and Twist promoter reporter for luciferase assay. As shown in Fig. 4, Luciferase activity was significantly increased in cells with HDV whole genome expression and cells with HDV-HBV co-expression. In contrast, if the SBE sites were lost (mt-pXP2-Twist), either HDV or HDV-HBV transfection could no longer induced Twist promoter activity.

### Statins inhibit L-HDAg activity on twist promoter

**Fig. 5:**
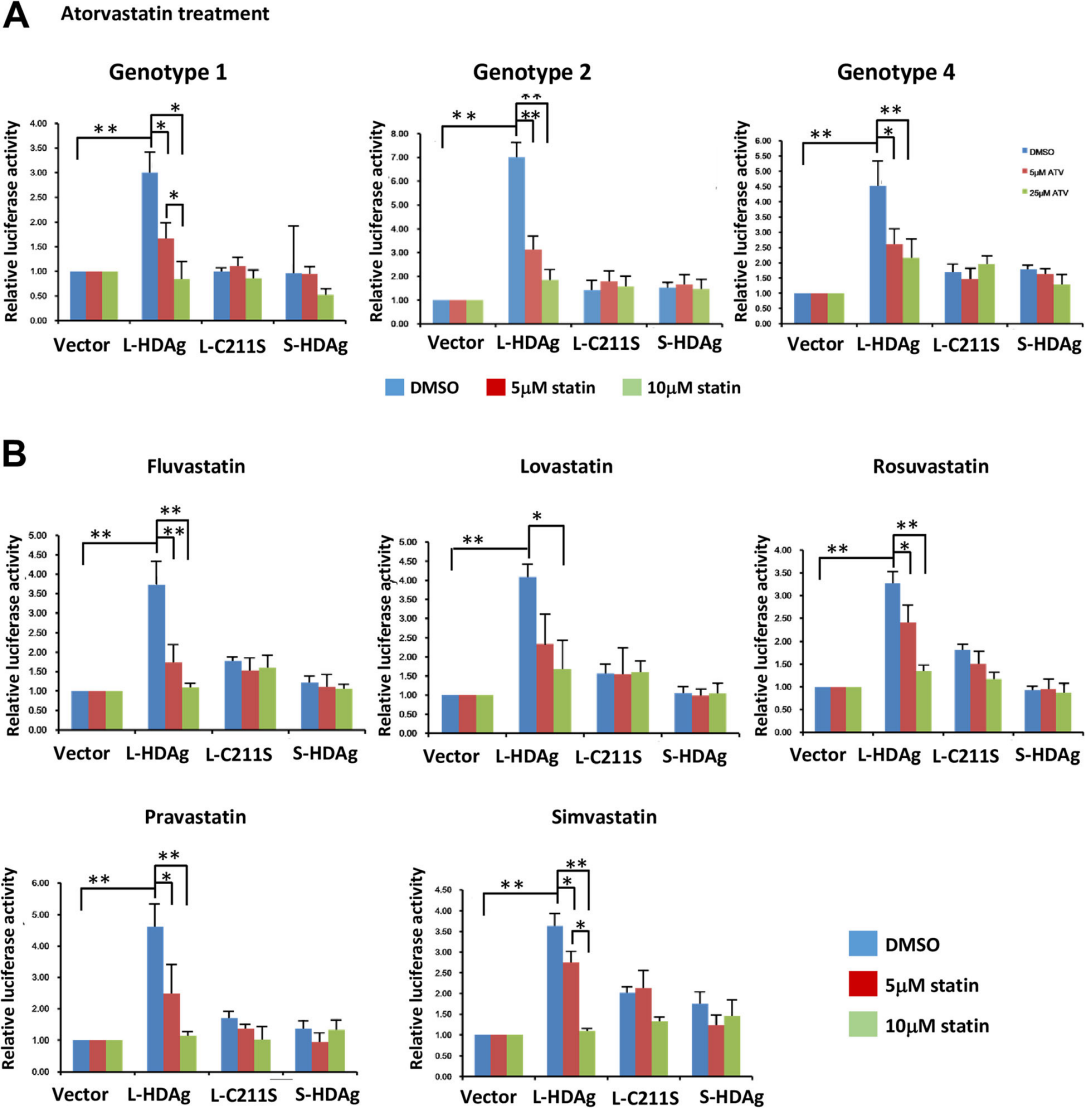
Statins suppress Twist promoter activity by indirectly reducing L-HDAg prenylation. **a** The pXP2-Twist reporter was co-transfected into Huh7 cells with L-HDAg, L-C211S, S-HDAg, or pcDNA3 control vector and treated with 5 or 25 μM atorvastatin for 72 h. Luciferase activities were measured and normalized relative to pCMV-β gal activity value. L-HDAg of all genotypes lost the ability to activate Twist promoter following atorvastatin treatment. Vector, L-C211S, and S-HDAg were negative controls and did not activate Twist promoter. Results shown are based on four independent experiments. *: *p* < 0.05; **: *p* < 0.01. **b** Inhibitory effects of five other statins on Twist promoter activity. Cells were transfected as in (**a**), and then treated with 5 or 25 μM fluvastatin, lovastatin, pravastatin, rosuvastatin, and simvastatin. Luciferase activities were measured. Results shown are based on five independent experiments. *: *p* < 0.05; **: *p* < 0.01

To test the hypothesis that statin treatment inhibits HDAg prenylation and results in effects similar to those observed for HDAg C211S mutant, we transiently co-transfected *Twist* promoter reporter of Huh7 cells with WT or C211S mutant of L-HDAg, and treated the transfectants with 5 or 25 μM atorvastatin. Luciferase activities were measured after 72 h treatment. For each of the three HDV genotypes, L-HDAg no longer induced *Twist* promoter activity following atorvastatin treatment (Fig. 5a). These findings further indicate that prenylation of cysteine residue of L-HDAg is essential for Smad3-mediated Twist activity.

Effects on *Twist* promoter expression by the other five commonly prescribed statins (lovastatin, simvastatin, pravastatin, rosuvastatin, fluvastatin) were also examined. In a previous study by G. Sass’s group, statin treatment at concentrations ranging from 1 to 100 μM had no toxic effect on Huh7 cells, except for a slight reduction of viability by fluvastatin and lovastatin [25]. We used concentrations 5 and 25 μM to evaluate the effects of the five statins on *Twist* promoter activity. Huh7 cells were transfected with L-HDAg-expressing plasmid, treated with statins after 6 h, and subjected to luciferase reporter assay at 72 h. Each of the five statins significantly reduced *Twist* promoter activity at concentration 25 μM (Fig. 5b). For simvastatin, the data had relatively smaller variability, and displayed a dose-dependent effect. Neither S-HDAg nor L-HDAg C211S mutation had a notable effect on *Twist* promoter activity relative to vector control.

### Simvastatin inhibits TGF-ß secretion and EMT markers expression

**Fig. 6:**
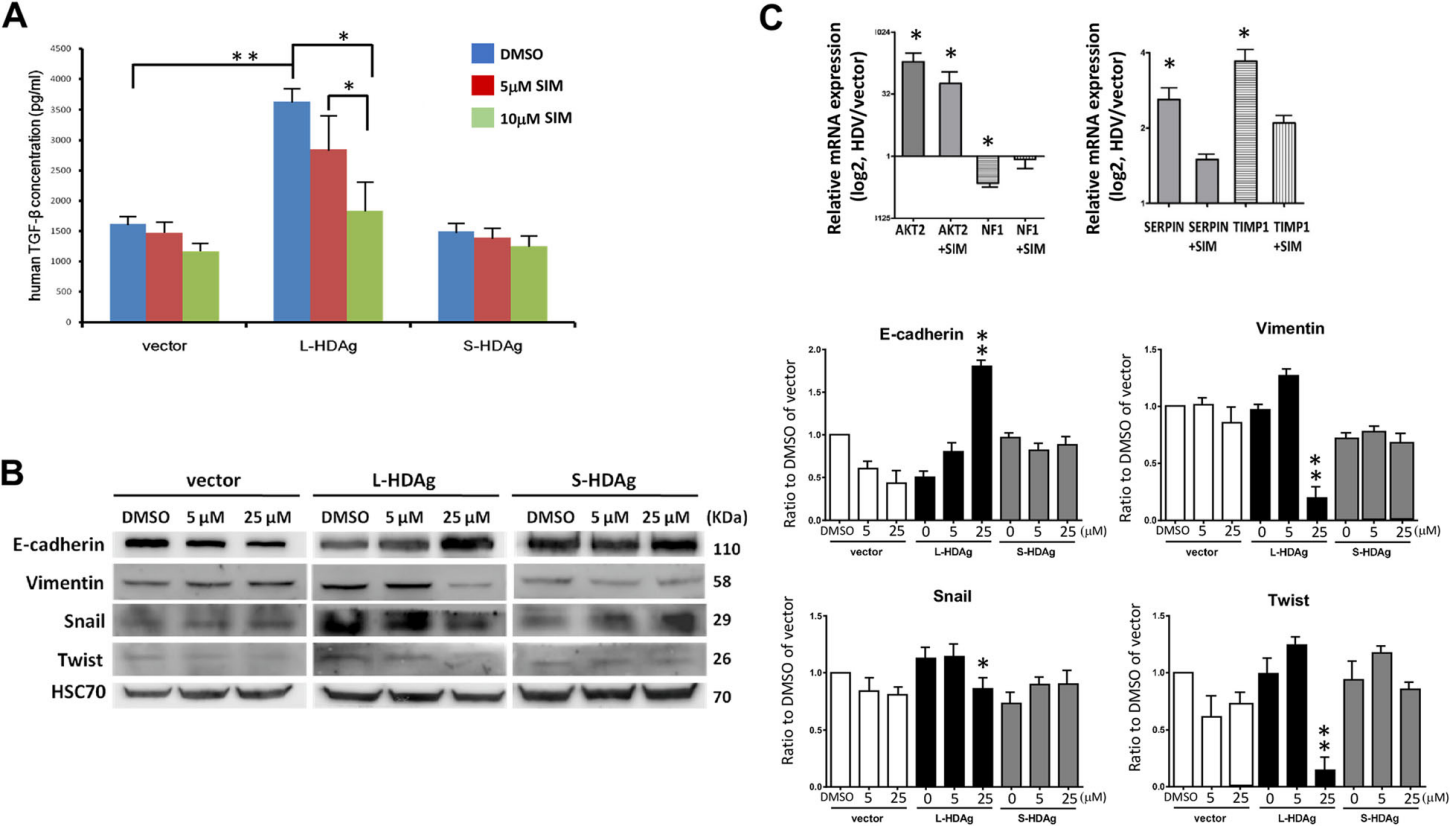
Simvastatin suppresses TGF-β secretion and EMT phenotype. **a** Huh7 cells were transfected with control vector (pcDNA3.1)-, L-HDAg-, or S-HDAg-expressing plasmids, and treated with various concentrations of simvastatin. Cells were incubated 3 days with low level of FBS (2%), and TGF-β level in supernatant was measured by ELISA. Results shown are based on four independent experiments. *: *p* < 0.05; **: *p* < 0.01. **b** Cells were transfected as in (**a**), and then treated with simvastatin. After 9 days, levels of EMT markers, such as Twist, Snail, E-cadherin, and vimentin were analyzed by Western blotting. Hsp70 was used as internal control. Representative Immunoblot images are based on three independent experiments. Results shown are based on five independent experiments. *: *p* < 0.05; **: *p* < 0.01 (compared to DMSO control in the same group). **c** The HDV L-HDAg or control vector plasmids were transiently transfected into Huh7 cells and treated with 25 μM simvastatin (SIM) for 9 days. The mRNA levels of Twist target genes (*AKT2* and *NF1*) and fibrosis marker genes (*Serpin1* and *TIMP1*) were analyzed by RT-QPCR. Results shown are mean ± SD from five independent experiments. *: *p* < 0.05; **p < 0.01 (compared with controls without simvastatin treatment)

Degree of TGF-β secretion was higher in L-HDAg-expressing Huh7 cells than in S-HDAg-expressing cells, or in cells transfected with vector control plasmid (Fig. 6a). To evaluate effects of simvastatin on TGF-β secretion, cells were transfected with L-HDAg-expressing, S-HDAg-expressing, or vector control plasmids under minimal FBS concentration, and treated with various dosages of simvastatin for 3 days. Culture supernatants were collected, and TGF-β was quantified by ELISA. Simvastatin at concentration 25 μM significantly (*p* < 0.05) reduced TGF-β secretion by L-HDAg-expressing cells (Fig. 6a, middle). In contrast, simvastatin treatment did not notably alter TGF-β secretion by cells transfected with S-HDAg-expressing plasmid (Fig. 6a, right) or vector control plasmid (Fig. 6a, left).

To further investigate effects of statins on EMT, we transfected Huh7 cells with HDAg-expressing plasmids or vector control plasmid, treated these cells with simvastatin for 9 days, and then analyzed levels of Twist, Snail, E-cadherin, and vimentin by Western blotting. EMT phenotype (upregulation of Twist, Snail, and vimentin; downregulation of E-cadherin) was enhanced in L-HDAg-expressing cells, but not in S-HDAg-expressing cells, relative to control (Fig. 6b). The density analysis of blots signals indicated that Simvastatin treatment, especially at high dosage (25 μM) for 9 days, resulted in suppression of EMT phenotype (i.e., downregulation of Twist, Snail and vimentin; upregulation of E-cadherin) (Fig. 6b). These findings indicate that statin treatment inhibited L-HDAg-mediated TGF-β secretion and EMT.

TGF-β is a central mediator of fibrogenesis. The effect of HDAg on Twist downstream genes and TGF-β relative fibrosis genes were investigated. It has been reported that Twist binds to *Akt2* promoter and enhances its transcriptional activity [12]. Twist was also considered to suppress *NF1* tumor suppressor gene and contribute to tumorigenesis [25]. As shown in upper panel of Fig. 6c, the overexpression of L-HDAg in Huh7 cells indeed resulted in a significant increase in *Akt2* expression and a decrease in *NF1* expression. These results further confirmed the effects of L-HDAg on transactivation of *Twist*. On the other hand, we found that overexpression of L-HDAg resulted in upregulation of fibrosis promoting genes *Serpin1* and *TIMP1*, suggesting that HDAg expression may promote liver fibrosis (Lower panel of Fig. 6c). The administration of Simvastatin offset the effects of L-HDAg on the downstream genes of Twist (Fig. 6c).

### Statin suppresses HDV assembly/release in vitro

Huh7 cells were co-transfected with whole genomes of HDV-expressing and HBsAg-expressing plasmids, and then treated with 5 or 25 μM simvastatin for 9 days. Cell transfectants were lysed, virus-like particles in culture medium were pelleted by ultracentrifugation, and intracellular and extracellular protein levels of HDAg were analyzed by Western blotting.

**Fig. 7:**
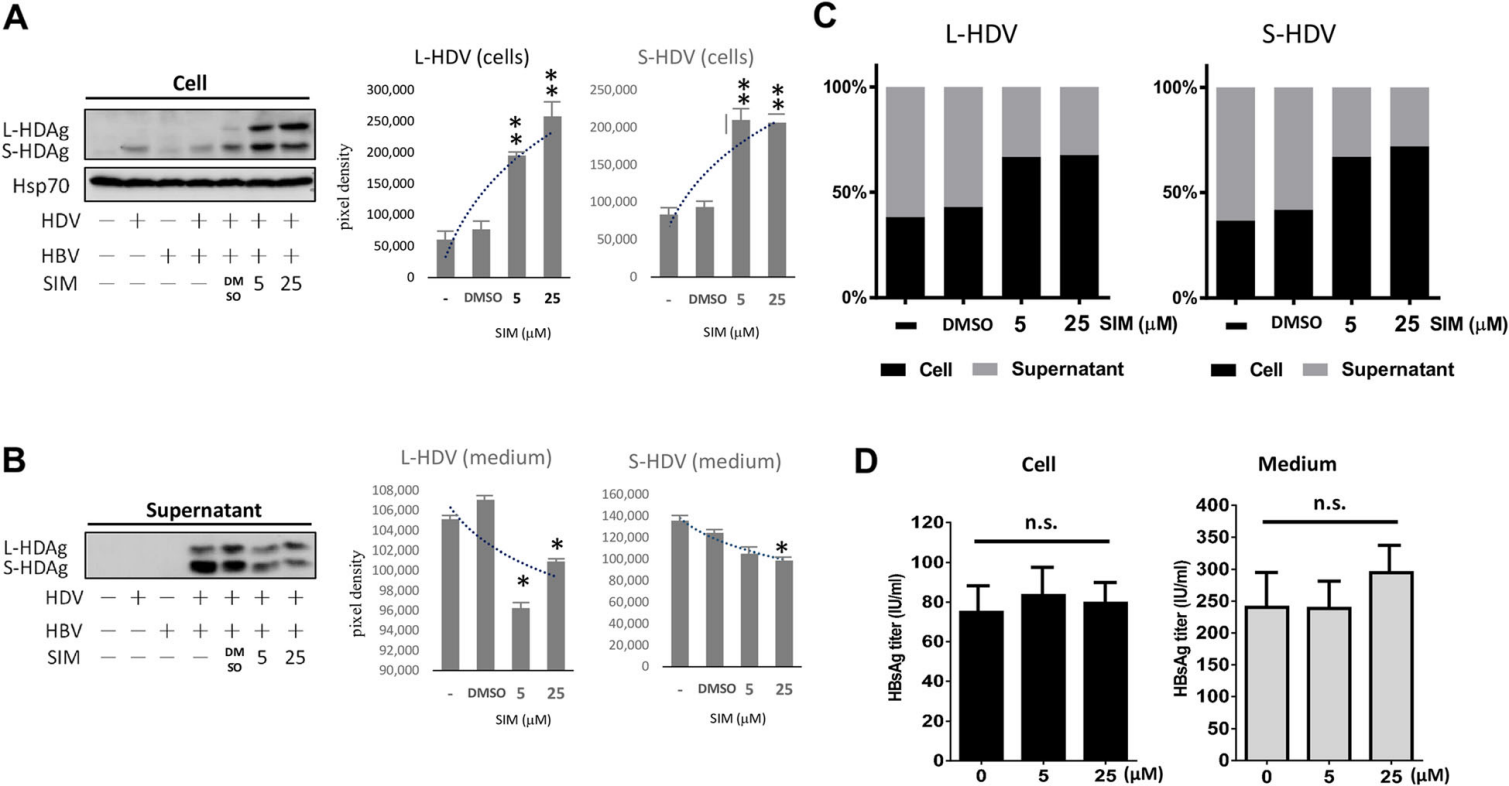
Simvastatin treatment suppresses HDV assembly and release. Huh7 cells were co-transfected with HBV- and HDV-expressing plasmids for 24 h and then treated with simvastatin (SIM, 5 or 25 μM) or DMSO (0 μM) for 9 days. The cells (**a**) and culture supernatants (**b**) were collected for Western blotting analysis of HDAg. Hsp70 was used as internal control. Signals corresponding to L-HDAg or S-HDAg were quantified by ImageJ and checked by Alpha Imager 3400. Results shown are based on three independent experiments. Dotted trend line indicates trend of change of L-HDAg or S-HDAg as a function of simvastatin (SIM) concentration. **c** Stacked bar graph showing distribution percentages of HDAg in cells and culture supernatants. The total HDAg in the cells and supernatant was defined as 100%. **d** The expression of HBsAg in cell or culture medium were analyzed by ELISA. *: *p* < 0.05; **p < 0.01 compared with DMSO control

**Fig. 8:**
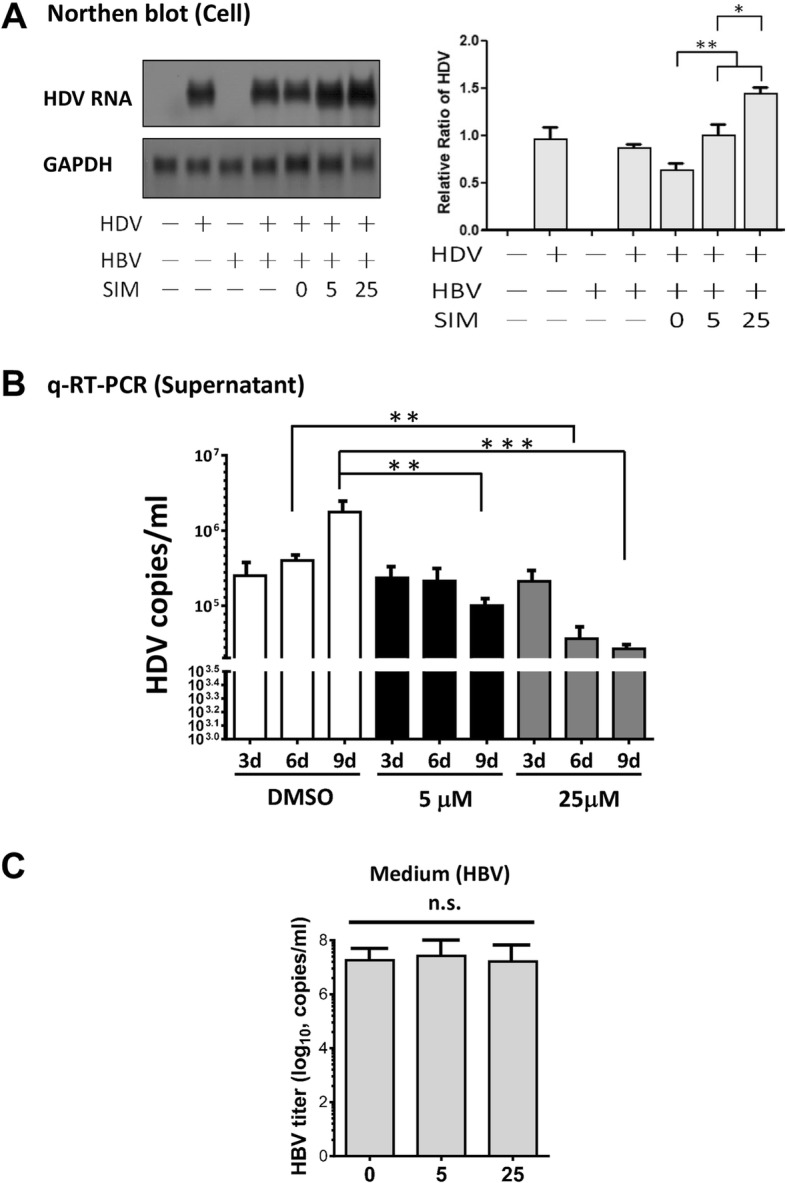
Treatment of statin lead to the retention of HDV RNA in cells. Northern blot assay (**a**) and q-RT-PCR analysis (**b**) were performed to evaluate the intracellular level and released HDV RNA levels in medium after simvastatin treatment in HBV/ HDV co-expressing Huh7 cells. After simvastatin treatment, HDV RNA retention in Huh7 cells increased significantly, secreted HDV RNA levels in medium were also significantly decreased in as compared to those of the DMSO controls. **c** The HBV DNA levels in culture medium were analyzed by Real-time PCR. **: *p* < 0.01, ***: *p* < 0.001

Protein levels of both L-HDAg and S-HDAg following simvastatin treatment were significantly elevated in cells (Fig. 7a), but reduced in medium (Fig. 7b). Distribution of HDAg was assessed by calculating percentages of intracellular and extracellular HDAg relative to total HDAg. Percentage of HDV released into medium was reduced by simvastatin treatment, whereas percentage of HDV remaining in cells increased in dose-dependent manner (Fig. 7c). In addition, Northern blot assay and q-RT-PCR were performed to evaluate the intracellular levels and medium released levels of HDV RNA after simvastatin treatment in HBV/ HDV co-expressing Huh7 cells. As shown in Fig. 8, after simvastatin treatment, the intracellular retention of HDV RNA in Huh7 cells increased significantly (Fig. 8a). In contrast, the release of HDV RNA were inhibited during the 9 days of simvastatin treatment (Fig. 8b). This result further supported that the treatment of statin impaired the assembly/release of HDV and lead to the accumulation of HDV RNA in cells. The whole genomes of HDV-expressing plasmid and HBV-expressing plasmids was co-transfected into Huh7, the effect of statin on HBV protein synthesis, HBV virus replication and secretion were analyzed by ELISA and Real-time PCR method. As shown in Fig. 7d and Fig. 8c, intracellular and extracellular HBV protein levels and HBV DNA levels in culture medium were unaffected by statins treatment.

**Fig. 9:**
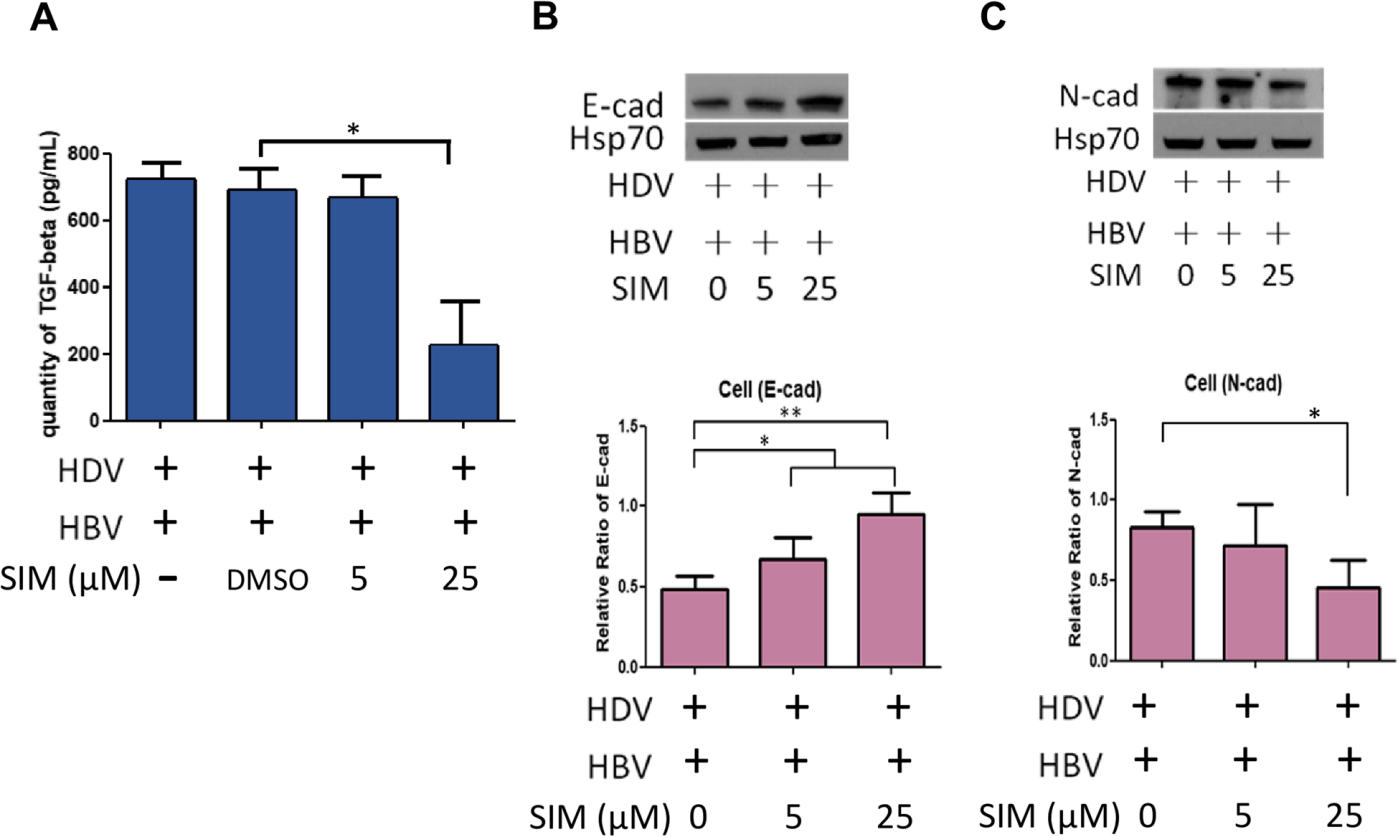
Simvastatin treatment suppresses TGF-β secretion and EMT phenotype in HBV/HDV-co-transfected Huh7 cell model. Cells were transfected with HBV- and HDV-expressing plasmids, and then treated with 5 or 25 μM simvastatin in 2% FBS medium (**a**) After 3 days of statin treatment, TGF-β levels of supernatants were analyzed by ELISA. TGF-β secretion was notably reduced by high-dose (25 μM) simvastatin treatment. **b**-**c** After 9 days of statin treatment, levels of E-cadherin and N-cadherin were analyzed by Western blotting. Hsp70 was used as internal control. Results shown are based on three independent experiments. *: *p* < 0.05; **: *p* < 0.01

To investigate effects of L-HDAg induction on TGF-β secretion, we co-transfected HBV and HDV-expressing plasmids into Huh7 cells, and treated these cells with 5 or 25 μM simvastatin. TGF-β secretion was increased in the co-transfected cells, but was then reduced by simvastatin treatment (particularly 25 μM simvastatin) (Fig. 9a). Possible reduction of EMT in relation to decreased TGF-β secretion was examined in HBV/HDV-co-transfected, simvastatin-treated cells as described above. In 25 μM simvastatin treatment, E-cadherin was upregulated significantly in these cells (Fig. 9b), whereas N-cadherin were downregulated (Fig. 9c). In summary, our experiments with a HBV/HDV co-transfection cell model indicated that statin treatment caused suppression of HDV virion release, TGF-β secretion, and EMT.

## Discussion

Chronic hepatitis D causes fulminant hepatic failure and liver cirrhosis, which may lead to HCC. Large delta antigen (L-HDAg) activates TGF-β which in turn induce EMT that may contribute to liver fibrosis and cirrhosis. L-HDAg can also induce ROS, STAT3 and NF-kappa B [12]. Of note, results of the present study reveal that L-HDAg of HDV binds to Smad3 and activates *Twist* gene expression through Smad3 binding element (SBE) in the proximal *Twist* promoter region. Mutations of the SBE or knockdown of SMAD3 markedly reduced the effects of L-HDAg on Twist promoter indicating the activation is specific. The findings that S-HDAg and L-HDAg cysteine mutant could not activate Twist promoter indicating that prenylation of L-HDAg is essential for Twist activation. Prenylation of L-HDAg on cysteine residue 211 is essential for transcriptional activation of *Twist* gene. Furthermore, the overexpression of L-HDAg resulted in a significant increase in *Akt2* expression, decrease in *NF1* expression and upregulation of Twist downstream fibrosis promoting genes *Serpin1* and *TIMP1*. This study not only found that L-HDAg has been reported to bind and activate SMAD3 consistent with the previous report [12], but also reported for the first time that L-HDAg can activate Twist through its interaction with SMAD3 and further contribute to EMT and the subsequent liver fibrosis.

In our previous study [10], selection of a novel dominant HDV strain with active HDV replication and secretion was associated with higher EMT activity and fluctuating course while selection of a novel dominant HDV variant with less active HDV replication and secretion was associated with low EMT activity and a gradual remitting course. In addition to necroinflammation induced by HBV and HDV infections, the findings of the currently study indicate that the activation of Twist and TGF-β via the specific binding of L-HDAg and SMAD3 may contribute to EMT and the accelerating liver fibrosis that appears to be a characteristic finding in HDV infection [2, 3, 26]. These findings provide a rationale of developing prenylation inhibitor to reduce HBV assembly and secretion in CHD patients.

There has been no very effective therapy for chronic hepatitis D, most of interferon therapy only has 15–36% sustained virological response rate [16]. Prenylation inhibitors were reported to suppress HDV secretion in vitro, however, HDV and L-HDAg appeared to be retained in cells [17, 18]. Recently, clinical trials of prenylation inhibitor in patients with CHD showed decrease of serum HDV RNA, however, serum HDV RNA and ALT returned to pre-treatment levels after discontinuation of therapy and many of them were intolerable to the combination of Lonafarnib 200 mg or 300 mg twice daily with pegylated IFN-α. However, the reduction of serum HDV RNA was only around 50% and the intrahepatic HDV and HBV replicating status was not clear. Moreover, side effects appeared frequent [27–29]. The combination of Lonarfarnib with Ritonavir showed better response and lower side effects [28, 29], however, the responses of HDV RNA and ALT had not lasted after discontinuation of therapy. Safety issue of long-term use is a concern [27–29]. HBV entry inhibitor and nucleic acid polymer appeared to be another novel therapy, however, long-term efficacy and side effects remain to be determined [29].

A group of drugs termed statins (inhibitors of HMG-CoA reductase) reduce biologically intermediate substrates for prenylation, and are widely used for reduction of cholesterol levels with low incidence of side effects. Recently, it was associated with the reduction of the risk of cirrhosis and its decompensation in CHB patients [30, 31] and the risk of HCC [32]. In the current study, treatment with statins reduced TGF-β secretion, EMT activity, levels of mesenchymal markers, and HDV release in vitro*.* Suppression of TGF-β and EMT by statin treatment presumably may contribute to subsequent prevention or reduction of liver fibrosis and HCC [12, 27–29]. This study provides mechanical basis for the beneficial effects of statins on the reduction of liver cirrhosis and HCC. It has been reported that L-HDAg is crucial for HDV assembly but it inhibits HDV replication [4, 33]. The inhibition of HDV on HBV replication has been reported in vitro and in human HDV superinfection [3, 34, 35]. Theoretically, retention of L-HDAg may suppress HDV and HBV replication. However, intrahepatic HDV replication or L-HDAg expression were not decreased in a limited time of statin administration of 9 days despite of the reduction of the assembly and secretion of HDV virions in this study consistent with previous reports [17, 18]. Hydrodynamic injection of HDV plasmid to tail vein of HBV-transgenic mice results in secretion of HDV virions and statin use reduced serum HDV virions [36]. However, this model does not allow the secreted HDV virions to infect neighboring hepatocytes mimicking human HDV infection. And intrahepatic HDV replication appeared not reduced in the study period [36] . In future study, hu-FRG mice which has chimeric human liver and allows HBV and HDV infection [37] will be inoculated with HBV and HDV to investigate the effects of statin treatment on HDV/HBV infection in vivo. The hu-GRG mice are more mimicking human condition. Statin treatment in this model can observe the effects of statin on HBV and HDV replication, assembly, secretion and the effects of reduced HDV viremia on the spread of HDV to neighboring hepatocytes. In our unpublished results, Simvastatin treatment reduced HDV viral load to 20% of pretreatment level. The longterm effects of statin treatment with or without the combination of nucleos(t) ide analogues on HBV and HDV viremia as well as intrahepatic HDV and HBV replication need further evaluation using longer treatment in larger number of HDV-infected FRG mice with untreated controls.

## Conclusion

**Fig. 10:**
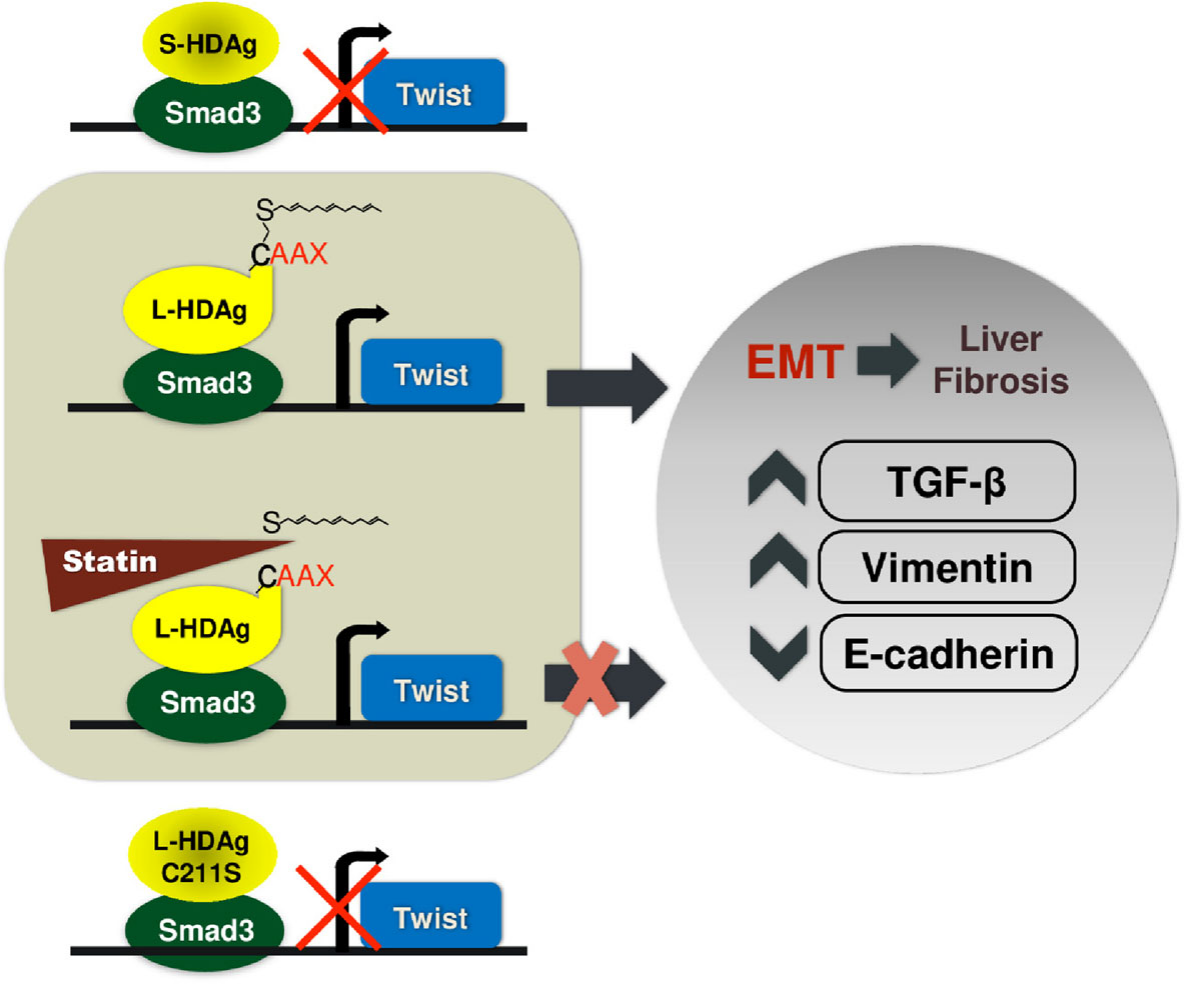
Schematic diagram illustrates that L-HDAg, but not S-HDAg, specifically activated the twist promoter through interact with Smad3. L-HDAg activates the expression of Twist, leading to increased EMT and TGF-β, and subsequent liver fibrosis. Statins inhibit the prenylation of L-HDAg, resulting in decreased expression of Twist, decreased secretion of TGF-β, and attenuate EMT, which can inhibit subsequent liver fibrosis. However, HDAg that cannot be prenylated, such as S-HDAg and L-HDAg C211S, cannot activate the Twist promoter

In this study, we found that L-HDAg of HDV specifically activates the Twist promoter through its interaction with Smad3. After activating the Twist promoter, L-HDAg induced TGF-β expression and EMT, and may further promote liver fibrosis (Fig. 10). Treatment with statins, a prenylation inhibitor, not only resulted in decreased *Twist* promoter activity, TGF-β expression, and EMT, but also significantly reduced the release of viral particles. The results of this study help clarify the mechanism of HDV-induced EMT and its relation to fibrosis, and provide a basis for novel therapeutic strategies against chronic hepatitis D infection. In addition, statin therapy for drug repositioning to reduce the risk of EMT, liver fibrosis and HCC may merit further study.

## Data Availability

Data and materials related to this study are available from the corresponding author on reasonable request.
